# Vision in schizophrenia: why it matters

**DOI:** 10.3389/fpsyg.2015.00041

**Published:** 2015-02-05

**Authors:** Steven Silverstein, Brian P. Keane, Randolph Blake, Anne Giersch, Michael Green, Szabolcs Kéri

**Affiliations:** ^1^Department of Psychiatry, Robert Wood Johnson Medical School, and University Behavioral Health Care, Rutgers, The State University of New JerseyPiscataway, NJ, USA; ^2^Department of Psychology, Vanderbilt UniversityNashville, TN, USA; ^3^Department of Psychiatry, University of StrasbourgStrasbourg, France; ^4^Department of Psychiatry and Biobehavioral Sciences, University of California at Los AngelesLos Angeles, CA, USA; ^5^Department of Physiology, University of SzegedSzeged, Hungary

**Keywords:** schizophrenia, vision, perception, risk, cognition, blindness, brain

Visual processing impairments are now well established in schizophrenia, including abnormalities in: contrast sensitivity (Kiss et al., 2010; Kelemen et al., 2013); excitatory and inhibitory functions such as those involved in forward and backward masking (Green et al., 2011) and surround suppression (Dakin et al., 2005); perceptual organization (Silverstein and Keane, 2011a); facial emotion recognition (Turetsky et al., 2007) and motion processing (Chen, 2011). There has been little work on color processing to date, but clinical reports indicate frequent descriptions of increased intensity of, or change in colors, in addition to changes in brightness contrast (Vollmer-Larsen et al., 2007). Of etiological relevance, visual distortions (which occur in over 60% of patients) have the highest sensitivity for conversion to a psychotic disorder among all basic symptoms (Klosterkotter et al., 2001). In addition, visual impairments in children of parents with schizophrenia predict later development of the disorder (Schiffman et al., 2006), and visual abnormalities in children in the general population are more strongly associated with the later development of schizophrenia than any other form of sensory impairment (Schubert et al., 2005). Finally, seemingly subtle visual impairments contribute to poorer real-world functioning (Rassovsky et al., 2011; Green et al., 2012). In short, visual changes (e.g., distortions, hallucinations) are common, and they have etiological, pathophysiological, and functional significance. In some cases, they can be viewed as models of impaired neural circuitry that can inform our understanding of the same connectivity problems occurring at larger scales, such as in the frontal lobe, or involving connections between brain regions (Phillips and Silverstein, 2003).

Given this, and the fact that vision is the most studied and best understood function in neuroscience, why is vision such an understudied area in schizophrenia research? (Silverstein and Keane, 2011b). Perhaps it is due to the misperception that visual findings are relatively unimportant aspects of the disorder. Much evidence, including that cited above, and included in this e-book, shows that to be untrue.

The 30 papers included in this volume make important contributions toward clarifying the mechanisms involved in visual impairments, and their relevance for schizophrenia. These are divided into sections on: (a) visual processing impairments in schizophrenia; (b) visual processing impairments in at-risk states, and the implications of data on an inverse relationship between congenital blindness and incidence of schizophrenia; and (c) broader theoretical papers. The first section begins with three papers on low-level visual impairments in schizophrenia, including findings on: (1) the interaction of color and contrast sensitivity effects (Cadenhead et al., 2013) (2) a bias toward low spatial frequency processing in face perception (Laprevote et al., 2013); and (3) the influence of comorbid PTSD on contrast sensitivity in schizophrenia. The next three papers consider inhibitory effects, including: (4,5) surround suppression reductions with a variety of stimuli (Tibber et al., 2013; Yang et al., 2013); (6) the effects of change in clinical status on size contrast (Silverstein et al., 2013); (7) object-substitution masking (Wynn et al., 2013); and (8) a general approach to backwards masking impairment in schizophrenia (Herzog et al., 2013). The next four papers cover issues related to mid-level vision and perceptual organization. These include those on: (9) sex differences and clinical variables related to perceptual organization impairment in schizophrenia (Joseph et al., 2013); (10) an event-related potential marker of contour integration impairment (Butler et al., 2013); (11) neural oscillations and perceptual organization (Spencer and Ghorashi, 2014); and (12) a review of oscillatory activity and its relevance for understanding visual processing in schizophrenia. The next two papers (13,14; Christensen et al., 2013; Darke et al., 2013) address the issue of face processing abnormalities in schizophrenia. The final papers in this section cover topics related to motion processing, eye movements, temporal context processing effects and time perception. These include studies of: 15–17) biological motion perception in schizophrenia (Hastings et al., 2013; Kim et al., 2013; Spencer et al., 2013); (18–19) eye movement and scan pattern abnormalities (Delerue and Boucart, 2013; Sprenger et al., 2013); (20) visual and motor disorganization (Giersch et al., 2013b); (21) oscillatory markers of abnormal temporal context processing (Dias et al., 2013); and (22) the role of impaired temporal processing in visual processing abnormalities in schizophrenia (Giersch et al., 2013a).

The second section addresses issues related to risk and prevention. These include two papers on the nature of visual processing impairments in schizotypy (23,24; Bressan and Kramer, 2013; Ribolsi et al., 2013); and three papers (25–27) on the hypothesis that congenital blindness serves as a protective factor against schizophrenia, as well as the implications of these data for early cognitive-perceptual training in at-risk populations.

The third section addresses general issues. Skottun and Skoyles (2013) (28) call into question the view that the magnocellular pathway is disproportionally impaired in schizophrenia; they argue instead for a more generalized dysfunction in visual perception. The paper by Yoon et al. (2013) (29) demonstrates how studies of visual processes in schizophrenia can reveal abnormalities in general computational processes in schizophrenia. Finally, Phillips and Silverstein (2013) (30) argue that a general mechanism of context-sensitive gain control is basic to cognition and perception and is impaired in schizophrenia, which can account for many observed findings in the disorder.

Taken together, these papers provide a representative example of current work in the vision science of schizophrenia. Our hope is that this set of papers will be of use to those currently working in this field, and will stimulate others to investigate these issues. Findings addressing these questions would be of major benefit to the field of schizophrenia research, and would also inform the study of normal visual perception.

## Conflict of interest statement

The authors declare that the research was conducted in the absence of any commercial or financial relationships that could be construed as a potential conflict of interest.
